# Non-medical costs during the first year after diagnosis in two cohorts of patients with early rheumatoid arthritis, enrolled 10 years apart

**DOI:** 10.1007/s10067-016-3470-z

**Published:** 2016-11-10

**Authors:** Magnus Husberg, Thomas Davidson, Eva Hallert

**Affiliations:** Center for Medical Technology Assessment, Department of Medical and Health Sciences, Linköping University, SE-58183 Linköping, Sweden

**Keywords:** Cohort study, Disease activity, Early rheumatoid arthritis, Gender, Health services research, Non-medical costs

## Abstract

The aim of the present study was to calculate non-medical costs during year 1 after diagnosis in two cohorts of patients with early rheumatoid arthritis enrolled 1996–1998 and 2006–2009. Clinical data were collected regularly in both cohorts. Besides information about healthcare utilization and days lost from work, patients reported non-medical costs for aids/devices, transportation, formal and informal care. Formal care was valued as full labour cost for official home help (€42.80/h) and informal care from relatives and friends as opportunity cost of leisure time, corresponding to 35% of labour cost (€15/h). In both cohorts, only 2% used formal care, while more than 50% used informal care. Prescription of aids/devices was more frequent in cohort 2 and more women than men needed aids/devices. Help with transportation was also more common in cohort 2. Women in both cohorts needed more informal care than men, especially with personal care and household issues. Adjusting for covariates in regression models, female sex remained associated with higher costs in both cohorts. Non-medical costs in cohort 2 were €1892, €1575 constituting informal care, corresponding to 83% of non-medical costs. Total non-medical costs constituted 25% of total direct costs and 11% of total direct and indirect costs. Informal care accounted for the largest part of non-medical costs and women had higher costs than men. Despite established social welfare system, it is obvious that family and friends, to a large extent, are involved in informal care of patients with early RA, and this may underestimate the total burden of the disease.

Rheumatoid arthritis (RA) is a chronic and disabling disease, and the economic consequences of the disease are substantial for patients and for society [1–3]. Costs caused by healthcare utilization are high, but total costs have hitherto mainly been driven by loss of working capacity [4]. The introduction of biological drugs during the last decade has led to improved management of the disease but has also substantially increased direct costs, which are now predominantly driven by drug costs [5, 6]. It has been suggested that the severity of the disease is declining, possibly due to early diagnosis and early treatment [7]. A decline in incidence, especially in women has also been reported, as well as a shift towards higher age at onset of disease [8]. By contrast, others have reported an increase in RA-incidence in women and also an increasing prevalence of RA [9].

We have previously reported the development of disease activity and costs in a cohort of patients with early RA enrolled 1996–1998 (TIRA1, T1, acronym for ‘early intervention in RA’) [3]. A new RA-cohort (TIRA2, T2), was launched 10 years later, 2006–2009, and is currently followed, collecting clinical and health economic data, using similar instruments and questionnaires as in the 1996–1998 cohort [10]. All patients were assessed at baseline (time point of inclusion), after 3 months and after 6 months and at 1-year follow-up, by a team care unit with rheumatologist, physiotherapist, occupational therapist and nurse, similarly in both cohorts. No interventions were performed according to a study protocol, and drug treatment decisions in both cohorts were made by the physician’s preference after physical examination. Patients were also offered multiprofessional intervention when considered necessary. In addition, all patients were given the opportunity to participate in an educational programme carried out by the multiprofessional team.

During the first year after diagnosis, the total costs in the 1996–1998 cohort amounted to €15,868, direct costs accounting for 30% and indirect costs 70% [3]. In the 2006–2009 cohort, there was a shift towards increasing direct costs and decreasing indirect costs, with total costs remaining basically unchanged, €15,358, direct costs accounting for 37% and indirect costs 63%. Days in hospital decreased in T2 compared to T1, but costs for outpatient care and drugs increased. T2 patients had a substantially higher prescription of DMARDs including biologics, compared to T1 patients, and the total direct costs amounted to €5716 in T2 vs €4674 in T1. Sick leave was lower in T2 compared to T1, but disability pension was higher, resulting in basically unchanged total loss of productivity [10].

From a healthcare perspective, direct costs typically include costs for outpatient visits, hospitalization, surgery and drugs, while adopting a societal perspective, relevant non-medical costs such as costs for medical aids, assistive devices, transportation, formal and informal care, regardless payer, should also be included [11]. There is no general consensus on which perspective should be adopted, and in our previous reports, as well as in many other reports, non-medical costs have not been included in the cost calculations [12–14].

The aim of the present study was to calculate the non-medical costs during the first year after diagnosis in two similar cohorts of patients with early RA, enrolled 10 years apart, from basically the same catchment area.

## Patients and methods

### Patients

During 1996–1998, 320 patients with early (≤1 year) RA were recruited from 10 rheumatology units in Sweden, corresponding to a catchment area of >1 million inhabitants (TIRA1, T1, acronym for ‘early intervention in RA’). A similar cohort, TIRA2 (T2), was launched 10 years later, and 463 patients with early RA were enrolled 2006–09.

### Clinical assessments

Clinical and laboratory data was collected regularly in both cohorts. The 28-joint-count disease activity score (DAS28) was calculated [15], and patients reported pain on a VAS-scale and completed the Health Assessment Questionnaire (HAQ) [16]. Details of the study are described previously [3].

### Health economic questionnaire

Besides a baseline questionnaire with sociodemographic data including age, sex, marital status, educational level and employment status, patients in both cohorts were provided with health economic questionnaires every 6 months. The questionnaires were kept as diaries, and patients reported continuously all health care utilization and number of days lost from work. The health related quality-of-life instruments EQ-5D and EQ-VAS (0–100) were also completed [17]. In addition, patients also reported non-medical costs such as costs for aids and devices, transportation, formal and informal care. Aids and devices were divided into 4 subgroups: (1) aids for personal care and activities of daily living (ADL) and household appliances, (2) orthoses such as collars, splints, insoles and shoes, (3) canes and crutches and (4) work adaptation aids such as modified chairs and desks. Pricing of aids and devices were obtained from companies and suppliers providing these devices (www.gulare.com, http://plus.rjl.se). Transportation, regardless mode of travel, be it somebody driving or using public transportation, was reported as the patient’s expenses for the trips or as a calculation of the travel distance, using official tariffs for costs per kilometre (www.trafa.se, www.fardtjansten.sll.se). Formal care was valued as full labour cost for official community home help (€42.80/h) (www.skl.se), while cost for informal care from relatives and friends was valued as the opportunity cost of leisure time, corresponding to 35% of full labour cost (€15/h). The questionnaire in T2 was slightly revised compared to the questionnaire in T1, but basically similar questions were asked.

Healthcare in Sweden is tax-financed, and apart from minor co-payments, basically all medical and non-medical costs, except informal care, are included in the welfare system, with almost full coverage of healthcare services. Costs in the first cohort were calculated, using unit costs from 2001, inflation adjusted to 2013, using the Swedish Consumer Price Index (CPI), and in the second cohort, unit costs from 2009, inflation-adjusted to 2013 with CPI. All costs were converted to 2013 euros, using the average exchange rate in 2013, €1 = 8.6494 SEK (www.riksbank.se). Costs were calculated, applying a societal perspective, including all costs, regardless payer.

### Statistics

Data are presented using descriptive statistics. Continuous variables are reported as means with standard deviations (SD) and categorical variables as numbers and proportions. Differences were analysed by Student’s *t* test, chi-square test or Fishers exact test when appropriate. Multivariate linear and logistic regression analyses were performed to explain non-medical costs and utilization of non-medical care, adjusting for covariates in the two cohorts and in women and men separately. Level of significance was set at *p* < 0.05. All analyses were performed using IBM SPSS 20.0.

### Ethical considerations

All patients gave written informed consent to participation. The study protocol was approved by the local ethics committee in Linkoping (Dnr M 168-05).

## Results

### Patients

Complete health economic questionnaires were available in 276/320 (86%) patients, 68% women in T1 and in 340/463 (73.4%) patients, and 70% women in T2 at 1-year follow-up. There were no differences in age, educational level, marital status, levels of sick leave and disability pension between patients with health economic data and patients with missing data.

Patients included in the present study had similar clinical characteristics at inclusion, except for women in T2, being older and reporting more pain and higher EQ-5D. Women also had slightly longer education, 11 vs 10.6 years, but there were no differences in DAS28 and HAQ between the cohorts (Table 1).

**Table 1:** Baseline characteristics of T1 and T2 at inclusion and *p* value for differences between the cohorts, differences between women in T1 and T2 and differences between men in T1 and T2

	Total			Women			Men		
	T1	T2	*p*	T1	T2	*p*	T1	T2	*p*
	*n* = 276	*n* = 340		*n* = 187	*n* = 239		*n* = 89	*n* = 101	
Age (yrs)	56 (15)	59 (14)	0.019	54 (15)	58 (13)	0.013	60 (13)	62 (14)	0.481
Cohabiting (%)	73	73	0.978	70	71	0.961	78	78	0.979
Education (yrs)	10.6 (2.1)	11.0 (2.3)	0.025	10.7(2.1)	11.2 (2.3)	0.050	10.3(2.0)	10.6(2.1)	0.351
DAS28	5.3 (1.2)	5.1 (1.3)	0.102	5.3 (1.2)	5.2 (1.2)	0.305	5.3 (1.0)	5.0 (1.4)	0.154
HAQ (0–3)	0.9 (0.6)	1.0 (0.6)	0.094	0.9 (0.6)	1.0 (0.6)	0.090	0.8 (0.5)	0.8 (0.6)	0.715
Pain (VAS)	48 (25)	53 (25)	0.017	48 (24)	54 (24)	0.030	47 (26)	51 (26)	0.310
EQ5D (0–1)	0.59 (0.26)	0.55 (0.27)	0.107	0.60(0.25)	0.55(0.27)	0.044	0.57(0.27)	0.57(0.28)	0.966
EQ-VAS (0–100)	58 (19)	57 (21)	0.478	59 (20)	56 (21)	0.176	58 (19)	60 (20)	0.397

*DAS28* 28 joint disease activity score, HAQ Health Assessment Questionnaire, *VAS* visual analogue scale, *EQ-5D* EuroQol 5-Dimensions, *EQ-VAS* general health, visual analogue scale

### Utilization of non-medical care

Only 2% of the patients used formal care during the first year after diagnosis, with no difference between the cohorts. The use of informal care was, however, much larger. More than 50% of patients in both cohorts needed help from relatives and friends with various issues. The informal caregivers provided, by far, most help with household activities such as cleaning, cooking, washing clothes, shopping and carrying heavy items, but also help with personal care such as dressing, eating and help with bathing and showering. A number of patients also needed help with outdoor activities such as gardening and snow shovelling. More women than men needed informal care, especially concerning household activities, 56.7% women vs 23.6% men in T1 (*p* < 0.001) and 57.3% women vs 30.7% men in T2 (*p* < 0.001). In T2, more women needed help with personal care, 25.5% women vs 9.9% men (*p* = 0.001) (Table 2 and Table 3).

**Table 2:** Proportion of patients (%) using formal and informal care, transportation and aids and devices during the first year. Differences between the 2 cohorts as well as between women and between men in the 2 cohorts respectively

	Total		*p*	Women		*p*	Men		*p*
	T1	T2		T1	T2		T1	T2	
	*n* = 276	*n* = 340		*n* = 187	*n* = 239		*n* = 89	*n* = 101	
Formal care	2.2	2.1	0.921	2.7	2.1	0.754	1.1	2.0	1.00
Aids/devices total	18.5	38.8	<0.000	21.4	45.6	<0.000	12.4	22.8	0.062
ADL devices	8.7	19.1	<0.000	10.2	23.4	<0.000	5.6	8.9	0.386
Orthoses	9.4	28.8	0.001	10.7	33.5	<0.000	6.7	17.8	0.022
Walking aids	NA	4.1	–	NA	4.6	–	NA	3	–
Work adaptation	4.7	NA	–	6.4	NA	–	1.1	NA	–
Transportation	40.6	63.5	<0.000	42.2	63.6	<0.000	37.1	63.4	<0.000
Informal care	51.4	52.4	0.823	59.4	60.3	0.852	34.8	33.7	0.866
Personal care	19.9	20.9	0.770	19.3	25.5	0.126	21.3	9.9	0.029
Household	46.0	49.4	0.401	56.7	57.3	0.895	23.6	30.7	0.274

*ADL* activities of daily living, including devices for personal care and household, *NA* not applicable

**Table 3:** Proportion of patients (%) using formal and informal care, transportation and aids and devices during the first year. Differences between women and men in T1 and between women and men in T2

	T1		*p*	T2		*p*
	Women	Men		Women	Men	
	*n* = 187	*n* = 89		*n* = 239	*n* = 101	
Formal care	2.7	1.1	0.668	2.1	2.0	1.00
Aids/devices total	21.4	12.4	0.071	45.6	22.8	<0.000
ADL devices	10.2	5.6	0.211	23.4	8.9	0.002
Orthoses	10.7	6.7	0.293	33.5	17.8	0.004
Walking aids	NA	NA	–	4.6	3	0.776
Work adaptation	6.4	1.1	0.067	NA	NA	–
Transportation	42.2	37.1	0.414	63.6	63.4	0.968
Informal care	59.4	34.8	<0.000	60.3	33.7	<0.000
Personal care	19.3	21.3	0.684	25.5	9.9	0.001
Household	56.7	23.6	<0.000	57.3	30.7	<0.000

*ADL* activities of daily living, including devices for personal care and household, *NA* not assessed

Total prescription of aids and devices was more frequent in T2 compared to T1, 38.8 vs 18.5% (*p* < 0.001). Prescription of devices facilitating everyday activities of daily living (ADL) was consistently higher in women than in men. Women were also to a higher extent prescribed orthoses, splints and orthopaedic footwear. Only few patients used canes or crutches in T2. As the specific question about walking aids was not asked for in T1, comparisons cannot be made. On the other hand, workplace adjustments paid for by the employer was reported in T1, but not in T2. Such adjustments, like special chairs and height adjustable desks, were available for 4.7% of patients in T1, the vast majority women.

Costs for transportation were high in both cohorts, similarly in men and women, but the proportion of patients needing help with transportation was significantly larger in T2, 63.5 vs 40.6% in T1 (*p* < 0.001) (Table 2 and Table 3).

### Costs of non-medical care

Proportion of patients using formal care was similar in the two cohorts, and there was no significant difference in costs. Total prescription of aids and devices was higher in T2, but average total costs did not differ between the cohorts. A large amount of basic inexpensive assistive devices, such as grab bars, loop scissors and grippers, were prescribed to many patients in T2, and this lowered average costs.

Costs for orthoses were significantly higher in T2. Orthoses for wrists, elbows and hands were frequently prescribed as well as knee braces and foot orthoses. Orthopaedic custom-moulded insoles and pads were often prescribed early in the disease course, and in some cases, patients were prescribed orthopaedic shoes, when pads and insoles did not have sufficient effect. Prescription of canes or crutches was reported only in T2, but was only used by 14 patients, and accordingly, average costs were low. Thirteen patients in T1 were provided with various ergonomic aids, for instance, height adjustable desks, at the employer’s expense. Although this was limited to few patients, the individual cost for each patient was rather high. Costs for transportation did not differ between the cohorts. More patients in T2 needed help with transportation, but the travelled distances for T2 patients were shorter, and this lowered the average costs (Table 4 and Table 5).

**Table 4:** Non-medical costs (€, mean, SD) during the first year in T1 and T2. Differences between the two cohorts as well as between women and between men in T1 and T2, respectively

	Total		*p*	Women		*p*	Men		*p*
	T1	T2		T1	T2		T1	T2	
	*n* = 276	*n* = 340		*n* = 187	*n* = 239		*n* = 89	*n* = 101	
Formal care	20 (247)	148 (1591)	0.145	8 (49)	182 (1859)	0.202	45 (429)	67 (587)	0.772
Aids/devices total	62 (264)	74 (154)	0.504	73 (289)	87 (168)	0.523	41 (201)	43 (109)	0.936
ADL devices	29 (209)	20 (78)	0.486	32 (226)	24 (89)	0.621	21 (167)	10 (45)	0.528
Orthoses	14 (61)	47 (101)	<0.000	17 (71)	55 (104)	<0.000	8 (34)	29 (90)	0.041
Walking aids	–	7 (45)		–	9 (50)		–	4 (30)	
Work adaptation	20 (129)	–		24 (137)	–		12 (110)	–	
Transportation	68 (143)	95 (417)	0.303	72 (150)	110 (493)	0.304	61 (128)	60 (97)	0.967
Total^a^	151 (399)	317 (1965)	0.167	152 (353)	379 (2303)	0.183	147 (483)	170 (663)	0.786
Informal care	–	1575 (3240)		–	1989 (3717)		–	596 (1149)	
Total^b^	–	1892 (3991)		–	2368 (4599)		–	766 (1356)	

– not assessed

*ADL* activities of daily living, including aids/assistive devices for personal care and household

^a^Total non-medical costs excluding informal care

^b^Total non-medical costs including informal care

**Table 5:** Non-medical costs (€, mean, SD) during the first year in T1 and T2. Differences between women and men in T1 and between women and men in T2

	T1		*p*	T2		*p*
	Women	Men		Women	Men	
	*n* = 187	*n* = 89		*n* = 239	*n* = 101	
Formal care	8 (49)	45 (429)	0.239	182 (1859)	67 (587)	0.545
Aids/devices total	73 (289)	41 (201)	0.346	87 (168)	43 (109)	0.015
ADL devices	32 (226)	21 (167)	0.688	24 (89)	10 (45)	0.137
Orthoses	17 (71)	8 (34)	0.233	55 (104)	29 (90)	0.031
Walking aids	–	–		9 (50)	4 (30)	0.365
Work adaptation	24 (137)	12 (110)	0.477	–	–	
Transportation	72 (150)	61 (128)	0.554	110 (493)	60 (97)	0.312
Total^a^	152 (353)	147 (483)	0.913	379 (2303)	170 (663)	0.371
Informal care	–	–		1989 (3717)	596 (1149)	<0.000
Total^b^	–	–		2368 (4599)	766 (1356)	<0.000

– not assessed

*ADL* activities of daily living, including aids/assistive devices for personal care and household

^a^Total non-medical costs excluding informal care

^b^Total non-medical costs including informal care

Costs for informal care are presented separately, since the information on informal care differed between the cohorts. In T1, patients reported if they had used any informal help and if so, what kind of help, but no information on the extent of help, i.e. number of hours needed. In T2, similar information was available, but in addition, T2 patients also provided detailed information about the number of hours with help, they had received each week or month. Hence, the proportion of patients needing informal care is available for both cohorts, but costs could be calculated only for the T2 cohort. During the first year, in the T2 cohort, women needed help on the average 132 h compared to men, who needed only 40 h (*p* < 0.000). The costs for informal care in T2 amounted to €1575, which is 5 times higher than total costs for remaining non-medical costs in T2 (Table 4 and Table 5).

**Fig. 1:**
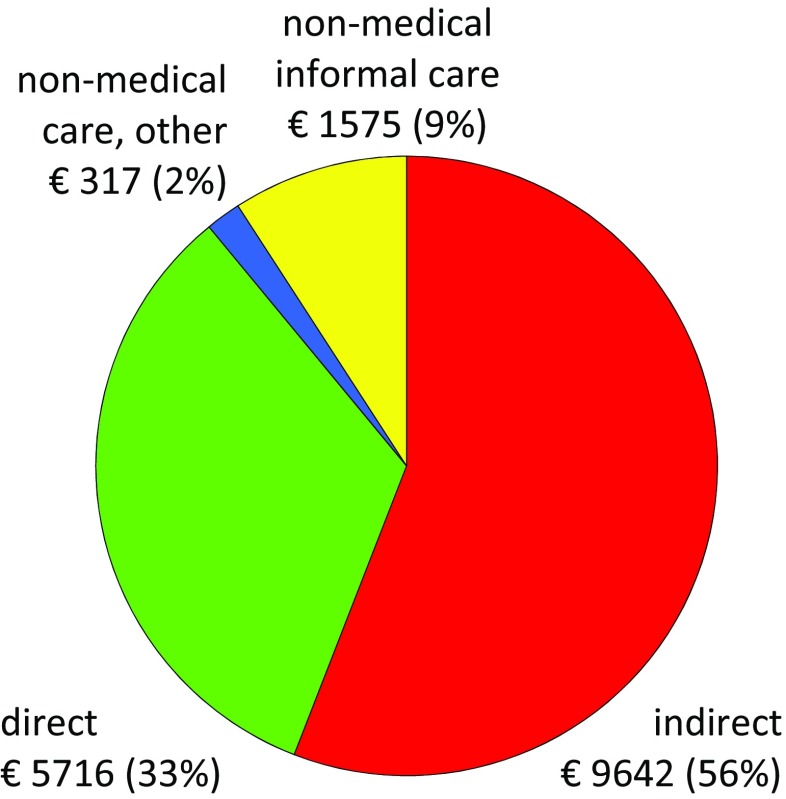
Direct costs, indirect costs and non-medical costs and proportions of costs. Non-medical costs are divided into costs for informal care and costs for other non-medical issues comprising formal care, aid/devices and transportation

The proportion of total non-medical costs, including informal care, amounted to 11% of total costs, with 9% accounting for informal care. The costs and proportions of costs for T2 patients are presented in Fig. 1.

In logistic and linear regression analyses, adjusting for age, gender, marital status, education, DAS28, HAQ, pain and EQ-5D, female sex was still significantly associated with higher costs for aids and devices and informal care in both cohorts and utilization of non-medical care was associated with female sex and cohort (data not shown).

## Discussion

Non-medical costs are often excluded in cost calculations, but for RA-patients, costs for medical aids, assistive devices, transportation, formal and informal care can be considerable [18]. In the present study, we have calculated non-medical costs in two cohorts of RA-patients, enrolled 10 years apart. In the latter cohort, T2, the non-medical costs, excluding informal care, were twice the size compared to the first cohort, T1. However, comparing these costs with previous calculations of direct and indirect costs in the 2 cohorts, the non-medical costs, excluding informal care, were small and had minor impact on the proportions, representing only 1 and 2% respectively of total costs [3, 10].

When costs for informal care were included in total non-medical costs, the proportions grew and increased from 2 to 11% of total costs and from 5% of direct costs to 25%. Since information on number of hours was available only in T2, costs for informal care could be calculated only in T2. However, since the proportion of patients using informal care was similar in both cohorts, 51.4 and 52.4%, respectively, we believe that costs for informal care in T1 are likely to be of the similar magnitude in T1 as in T2.

Direct comparisons between various studies are hazardous since healthcare systems differ between countries, depending on various social systems and availability of healthcare. Levels of remuneration differ, and availability of aids and devices as well as formal care can be limited. Bearing this in mind, some comparisons can still be made.

In a Belgian study, informal care, valued at €8.70/h, made up for 51% of total direct costs [19]. This is higher than in our study, even though our price per hour is higher (€15/h), but could be explained by the Belgian study, including informal care for all causes, while our costs were limited to RA-related costs. A French study reported that total non-medical costs made up for 29% of total direct costs [13] which is close to our 25%. The French study, however, valued informal care by the replacement method, using the hourly rate of formal home help, while in the present study, informal care was valued as the opportunity cost of leisure time, corresponding to 1/3 of cost for formal home help. In a study from Italy, Turchetti et al. [20] estimated that informal care made up for 84% of total non-medical costs and this is in line with our results, where informal care made up for 83% of total non-medical costs. In a systematic review, Lundkvist et al. reported that total non-medical costs in Sweden made up for 29% of direct costs, which is close to our 25 and 14% of total costs and this is also close to our 11% [21].

There are many difficulties in estimating the value of informal care, in both quantifying the number of hours used and valuing the hours. The main methods used for quantifying the time for informal caregiving are the diary method and the recall method. Comparative studies have shown that the recall method may give a higher estimate of informal care hours [22]. In addition, when the informal caregiver performs activities that benefit himself while providing informal care, joint production occurs and this should preferably be deducted from the cost of informal care. This information was, however, not available and could not be taken into account in the present study.

When estimating the value of an hour of informal care, there are different theoretical approaches, as well as a variety in how these approaches are applied. The opportunity cost method calculates the costs of informal care as the value of the best alternative use of the time used for informal care. If the caregiving hours could be used for formal (paid) production, the value of informal care is equal to the value of this production. If the informal caregiver uses his or her leisure time, the cost of informal care is equal to the value of this leisure time. An alternative to the opportunity cost method is the replacement cost method or shadow price, which means that the value of the informal care is given by the cost of hiring formal care, adjusting for different efficiency between informal and formal care [23]. In the present study, we have based the value estimate on the opportunity cost method, assuming that the informal care providers mainly used leisure time, making a rather conservative assumption that leisure time can be valued at 35% of full labour cost (€15/h). In a recent study, trying to attribute a monetary value of patients’ leisure time (not participating in the labour market), travel time and treatment time were valued at approximately €13/h, which is basically in line with our valuation [24].

Despite the various methods of calculating costs, costs for informal care often represent the largest part of total non-medical costs [21, 25]. In the present study, informal care had by far the largest impact on total non-medical costs and made up for costs that were 5 times higher than all other non-medical costs together. Women needed significantly more care with household issues in both cohorts and in T2, also more help with personal care. This could indicate that women were more affected by the disease, but regression analyses showed that female sex was associated with higher costs, independent of DAS28 and HAQ. HAQ was similar in men and women at inclusion, but despite substantial improvement for all patients, women had higher HAQ scores at all follow ups, and this could explain some of the differences [10]. There is also a possibility that women in some cases had the main responsibility for household issues and hence needed more help with those specific issues.

One might assume that non-medical costs could be lower in the 2006–2009 cohort, compared to the 1996–2008 cohort, due to more active treatment strategies in the recent decade [26]. This was, however, not the case. Most costs were similar, except costs for orthoses, which were significantly higher in T2. It remains challenging to evaluate if the increasing use of biologics will affect non-medical resource use over the following years [26, 27]. In the present study, biological drugs were available only for T2, but were prescribed to very few patients during the first year after diagnosis.

There are a number of limitations in the present study. The use of self-reported information from questionnaires may be biased by selective information. The questionnaires were however kept as diaries over the period, and the patients registered continuously all medical and non-medical healthcare utilization.

The T2 questionnaire was slightly revised compared to the T1, and this lead to some cost components being grouped differently in the cohorts. For instance, in T2, costs for canes and crutches were reported separately while in T1, these costs were included in costs for aids and devices. Costs for workplace adjustments paid for by the employer was reported in T1, but not in T2. Although only 4.7% of patients in T1 used this, the adjustments were rather costly and corresponding costs are probably not included in any other cost domain in the T2 questionnaire. Since workplace adjustments for employees are available to the same extent in 2006–2009 as in 1996–2008, we believe that patients in T2 were likely to have basically similar average costs paid for by the employer. However, if costs for work adaptation, for equal comparisons, are deleted from T1 (or the corresponding value is added to T2), the difference between the cohorts remains and costs in T2 increases further compared to T1.

Some patients were offered arrangements by the employer such as allowing rest periods during the day and changing the time that work started in the morning. These measures were difficult to quantify and have not been taken into account.

A strength of the present study is the well-characterized patient material and the longitudinal prospective design with regular follow-ups, allowing analyses of long-term outcomes in two cohorts of patients with early RA, included from basically the same catchment area 10 years apart. The bottom-up approach with questionnaires in our study is also important, since information on most non-medical costs is available only through patient-derived data.

To conclude, informal care accounted for the largest part of non-medical costs and women had higher non-medical costs than men, during the first year after diagnosis. Despite an established social welfare system, it is obvious that family and friends, to a large extent, are involved in informal care of patients with early RA, and this may underestimate the total burden of the disease for patient and for society.

## References

[1] Kalkan A, Hallert E, Bernfort L, Husberg M, Carlsson P (2014). Costs of rheumatoid arthritis during the period 1990–2010: a register-based cost-of-illness study in Sweden. Rheumatology.

[2] Furneri G, Mantovani LG, Belisari A, Mosca M, Cristiani M, Bellelli S, Cortesi PA, Turchetti G (2012). Systematic literature review on economic implications and pharmacoeconomic issues of rheumatoid arthritis. Clin Exp Rheumatol.

[3] Hallert E, Husberg M, Jonsson D, Skogh T (2004). Rheumatoid arthritis is already expensive during the first year of the disease (the Swedish TIRA project). Rheumatology.

[4] Lenssinck ML, Burdorf A, Boonen A, Gignac MA, Hazes JM, Luime JJ (2013). Consequences of inflammatory arthritis for workplace productivity loss and sick leave: a systematic review. Ann Rheum Dis.

[5] Eriksson JK, Johansson K, Askling J, Neovius M (2015). Costs for hospital care, drugs and lost work days in incident and prevalent rheumatoid arthritis: how large, and how are they distributed?. Ann Rheum Dis.

[6] Hallert E, Husberg M, Bernfort L (2012). The incidence of permanent work disability in patients with rheumatoid arthritis in Sweden 1990–2010: before and after introduction of biologic agents. Rheumatology.

[7] Uhlig T, Heiberg PM, Kvien TK (2008). Rheumatoid arthritis is milder in the new millennium: health status in patients with rheumatoid arthritis 1994–2004. Ann Rheum Dis.

[8] Uhlig T, Moe RH, Kvien TK (2014). The burden of disease in rheumatoid arthritis. PharmacoEconomics.

[9] Myasoedova E, Crowson CS, Kremers HM, Therneau TM, Gabriel SE (2010). Is the incidence of rheumatoid arthritis rising? Results from Olmsted County, Minnesota, 1955–2007. Arthritis Rheum.

[10] Hallert E, Husberg M, Kalkan A, Bernfort L (2016). Rheumatoid arthritis is still expensive in the new decade: a comparison between two early RA cohorts, diagnosed 1996–98 and 2006–09. Scand J Rheumatol.

[11] Drummond M, Sculpher M, Torrance G, O’Brien B, Stoddart G (2015). Methods for the economic evaluation of health care programmes.

[12] Hülsemann JL, Mittendorf T, Merkesdal S, Handelmann S, von der Schulenburg JM, Zeidler H, Ruof J (2005). Direct costs related to rheumatoid arthritis: the patient perspective. Ann Rheum Dis.

[13] Kobelt G, Woronoff AS, Richard B, Peeters P, Sany J (2008). Disease status, costs and quality of life of patients with rheumatoid arthritis in France: the ECO-PR Study. Joint Bone Spine.

[14] Heintz E, Gerber-Grote A, Ghabri S, Hamers F, Prevolnik Rupel V, Slabe-Erker R, Davidson T, EUnetHTA Joint Action 2, Work Package 7, Subgroup 3 (2016). Is there a European view on health economic evaluations? Results from a synopsis of methodological guidelines used in the EUnetHTA partner countries. PharmacoEconomics.

[15] Prevoo MLL, van’t Hof MA, Kuper HH, van Leeuwen MA, Van de Putte LBA, van Riel (1995). Modified disease activity scores that include twenty-eight-joint counts. Arthritis Rheum.

[16] Ekdahl C, Eberhardt KB, Andersson SI, Svensson B (1988). Assessing disability in patients with rheumatoid arthritis. Scand J Rheumatol.

[17] EuroQol G (1990). EuroQol—a new facility for the measurement of health-related quality of life. Health Policy.

[18] Krol M, Brouwer W (2015). Unpaid work in health economic evaluations. Soc Sci Med.

[19] Westhovens R, Boonen A, Verbruggen L, Durez P, De Clerck L, Malaise M, Mielants H (2005). Healthcare consumption and direct costs of rheumatoid arthritis in Belgium. Clin Rheumatol.

[20] Turchetti G, Bellelli S, Mosca M (2014). The social cost of rheumatoid arthritis in Italy: the results of an estimation exercise. Reumatismo.

[21] Lundkvist J, Kastang F, Kobelt G (2008). The burden of rheumatoid arthritis and access to treatment: health burden and costs. Eur J Health Econ.

[22] Van den Berg B, Spauwen P (2006). Measurement of informal care: an empirical study into the valid measurement of time spent on informal caregiving. Health Econ.

[23] Davidson T, Levin LA (2010). Is the societal approach wide enough to include relatives? Incorporating relatives’ costs and effects in a cost-effectiveness analysis. Appl Health Econ Health Policy.

[24] Van den Berg B, Gafni A, Portrait F (2013). Attributing a monetary value to patients’ time: a contingent valuation approach. CHE research paper 90. Centre for Health Economics.

[25] Mittendorf T, Dietz B, Sterz R, Cifaldi MA, Kupper H, von der Schulenburg JM (2008). Personal and economic burden of late-stage rheumatoid arthritis among patients treated with adalimumab: an evaluation from a patient’s perspective. Rheumatology.

[26] Fautrel B, Cukierman G, Joubert JM, Laurendeau C, Gourmelen J, Fagnani F (2016). Healthcare service utilisation costs attributable to rheumatoid arthritis in France: analysis of a representative national claims database. Joint Bone Spine.

[27] Verstappen SM (2015). Rheumatoid arthritis and work: the impact of rheumatoid arthritis on absenteeism and presenteeism. Best Pract Res Clin Rheumatol.

